# Research on efficiency measurement and spatiotemporal disparity of rural public health services in China

**DOI:** 10.1371/journal.pone.0252871

**Published:** 2021-07-22

**Authors:** Liqing Li, Zixuan Liu

**Affiliations:** College of Public Management & Law, Hunan Agricultural University, Changsha, Hunan, China; Institute for Advanced Sustainability Studies, GERMANY

## Abstract

**Objective:**

Based on the rural public health services of 29 regions in China, a service efficiency evaluation index system consisting of input and output dimensions was constructed, and the coupling, coordination and disparity efficiencies of rural public health services in China were studied, providing information to redress the imbalance in the interregional coordinated development.

**Methods:**

The efficiency, spatiotemporal disparity pattern, spatial correlation and evolutionary trend of the coordinated development of rural public health services of 29 regions in China from 2004 to 2018 were analyzed using efficiency and spatial analysis methods such as Data Envelopment Analysis (DEA), kernel density estimation and Moran’s Ⅰ analysis.

**Result:**

Nowadays, there are problems of unbalanced and insufficient development between the fields and regions in the development of rural public health services in China. The development level of rural public health services in various regions shows a distribution pattern that the service efficiency is “high in the middle”, “middle in the east” and “low in the west”, indicating a spatial cluster effect.

## Introduction and literature review

The increasing health needs and the impact of various public health events raise a great demand for paying attention to the efficiency and level of health services. In recent years, China’s health investment has continued to increase, and the level of basic health services has also been gradually improved. However, in general, the health services in the vast rural areas are still facing severe challenges, and the disparities in health services still exist between regions. In particular, the outbreak of Covid-19 epidemic caused a great shortage of health supplies in rural areas of China, and some provinces and cities with severe epidemic conditions were even faced with the shortages of health personnel and hospital beds, which poses a huge challenge to the national health system. Therefore, evaluating the allocation efficiency of rural health service resources in China’s provinces is a prerequisite for an effective response to the epidemic. The ability of China’s provinces to efficiently allocate the available health service resources is the key to resisting this epidemic. Hence, the evaluation of the efficiency and level of rural health services in various provinces will help to further improve the ability to support and allocate health service resources. In summary, a research on the efficiency measurement and spatiotemporal disparity of rural public health services in China is necessary.

The spatial disparities in the level of health services have been widely studied. Some studies have found that the provision of health services shows varying degrees of spatial imbalance in both developed and less developed countries such as the United States, Spain, Germany and India [1–5]. A number of studies have been conducted in recent years on the coordinated development of public health services in China. Lu Zuxun, Xu Hongbin et al,. [6] pointed out that although China’s health service system construction has made many achievements, the capacity of health services in grassroots is still insufficient. According to relevant studies, He Wenju and Liu Huiling [7] proposed that it is not uncommon for provinces, cities, and even regions to have different health resources. However, in recent years, the improvement of the system, the economic system and transportation environment has bridged the gap in health resources between provinces, cities and regions. In terms of index system construction, a series of variables were selected to construct the regional health service evaluation index system. Ma Zhifei et al,. [8] selected the number of skilled health personnel per capita and the number of beds in health facilities per capita as variables; Han Zenglin et al,. [9] selected the number of daily visits per doctor, daily inpatients per doctor and daily inpatients per health personnel as variables; Huang Jingnan et al,. [10] selected the number of doctors (assistant) per 1000 rural population and the number of registered nurses per 1000 rural population as variables. Based on this, an evaluation index system was constructed. Hu Yujie [11] used the DEA model and the Malmquist index to analyze the level of health service provision in various regions, and concluded that it is steadily increasing across China. Some authors also conducted a comprehensive evaluation by constructing a health evaluation index system. In general, multidimensional output indexes, such as the service capacity of grassroots health institutions and quality medical staff, are first selected to construct an evaluation index system [12]. The Gini coefficient, Theil index, entropy-weighted TOPSIS, concentration index method and other research methods are then used to measure the efficiency of public services [13–15]. It is generally agreed that regional disparities obviously exist between different regions and provinces in China, and the spatial pattern generally shows the distribution characteristics of “high in the east and low in the west” [16].

A review of the existing references reveals that the research on health service provision has made some progress in recent years. However, there are few references that focus specifically on the regional disparities in health service provision. The researches on the regional disparities in health service provision of China suffer from the following deficiencies: 1) The evaluation index systems are not well designed and comprehensive, and most of them lack results-based indexes, which may reduce the accuracy of the evaluation results; 2) Few studies have comprehensively and systematically discussed the distribution dynamics and regional disparities. More importantly, the lack of reasonable use of spatiotemporal analysis methods usually leads to an incomplete analysis and an inaccurate estimation result [17]. Therefore, based on previous studies and using China’s provincial panel data, this study analyzed the level of public health services in rural China from the perspectives of time and space. Firstly, the Data Envelopment Analysis (DEA) was used to quantitatively evaluate the level of health service provision in various regions of China from a perspective of time. Secondly, the kernel density estimation was used to compare different years and obtain temporal changes in the efficiency of health services in rural China. Then the Moran’s Ⅰ analysis was used to reveal the regional and spatial evolution of the level of health service provision. Hence, in this work, the efficiency of public health services in rural China was measured and studied in terms of time and space.

## Methodology

### 1. Selection of Data Envelopment Analysis (DEA) model

First of all, the purpose of the evaluation is to accurately know the current status of regional public health services. Therefore, the indexes that can reflect the effectiveness of rural public health services were selected. Based on the study of the index system and the summary of past experience, the cross-sectional data for one year were selected as input and output factors. The DEAP 2.1 software was used to calculate these data and obtain the evaluation value *θ* [18].

If there are n decision making units (DMU), each unit has m types of inputs and s types of output, which correspond to the “consumed resources” and “performance of work”. *X*_*ij*_(*X*_*ij*_>0; *i* = 1,2,⋯,*m*) refers to the input amount of the i-th input of the j-th decision unit, X_j_ is used to represent the output amount of the *DMU*_*j*_–th output of the j-th decision unit, and it is expressed as:

$$X_{j}={\left[x_{1j},x_{2j},\cdots \;x_{mj}\right]}^{T},Y_{j}={\left[y_{1j},y_{2j}\cdots \;y_{sj}\right]}^{T}\;(j=1,2,\cdots ,n)$$ (1)

(*X*_*j*_, *Y*_*j*_) can be used to denote the inputs and outputs of j decision units *DMU*_*j*_. After the introduction of the Archimedean infinitesimal ε, the input relaxation variable *S*^−^ and the output relaxation variable *S*^+^, the BCC model for the k-th decision unit is as follows:

$$\{\begin{array}{c} min[\theta -\varepsilon (\hat{e}S^{-}+e^{T}S^{+})]\\ \sum_{j=1}^{n}\lambda_{j}X_{j}+S^{-}=\theta X_{k}\\ \sum_{j=1}^{n}\lambda_{j}Y_{j}-S^{+}=Y_{k}\\ \sum_{j=1}^{n}\lambda_{j}=1\\ \lambda_{j}\geq 0;j=1,2,\cdots ,n\\ S^{+}\geq 0;S^{-}\geq 0 \end{array}$$ (2)

In the C^2^R model, θ is often referred to as the efficiency coefficient. If θ<1, but *S*^−^ and *S*^+^ are not all 0, the decision unit DEA under evaluation is considered inefficient, i.e., the existing outputs can be achieved with fewer inputs. If θ = 1 and one of *S*^−^ and *S*^+^ is not 0, the decision unit DEA is considered weakly efficient. If θ = 1 and *S*^−^ and *S*^+^ are both 0, the decision unit DEA is considered efficient, which means that it is not advisable to increase or decrease the input amount with the existing outputs. Comparing with the C^2^R model, the BBC model has an additional constraint, i.e., $\sum_{j=0}^{n}\lambda_{j}=1$. If the optimal solution to this model is λ*,S*^−^,S*^+^,θ*, its DEA efficiency is determined theoretically as follows:

1. When θ* = 1, the *j*_0_-th DMU is weakly efficient
2. When θ* = 1 and S*^−^ = 0, S*^+^ = 0, then the *j*_0_-th DMU is efficient.

Solve the above formula, if *θ* = 1, then the decision unit DMU_j_ being evaluated is DEA efficient. This result means that DMU_j_ reaches the maximum output with the set input or minimizes the input when achieving the set output target, and the resources have an efficient allocation. If *θ*<1, then the decision unit DMU_j_ being evaluated is not DEA efficient. This result means that there is insufficient output or excessive input in the evaluation system composed of these U decision units, indicating that the optimal allocation of resources has not been achieved.

### 2. Kernel density estimation

The kernel density estimation method is a non-parametric test method that studies the data distribution characteristics from the data sample itself to estimate unknown density functions. The principle is to count the number of points around a certain point. For data x1,x2,…,xn, the kernel density estimation is expressed as [19]:

$$f(x)=\frac{1}{Nh}\sum_{i=1}^{N}\text{K}(\frac{{\text{X}}_{i}-\bar{\text{X}}}{\text{h}})$$ (3)

Where K(x) denotes a Kernel function, Xi denotes the sample value of the random variable, $\bar{\text{x}}$ denotes the average value of the sample values, N is the sample size, h is the bandwidth. The choice of bandwidth directly affects the estimation results, such as the smaller the bandwidth the higher the accuracy of the estimation. In this work, the Gaussian Kernel density function was chosen among the commonly used Kernel functions, as shown in equation (4):

$$\text{K}(x)=\frac{1}{\sqrt{2\Pi }}exp(-\frac{x^{2}}{2})$$ (4)

### 3. Spatial global correlation analysis

Global correlation means the correlation of spatial data in the entire space and is measured by the global Moran’s I which is used to test whether neighboring areas in the entire space are similar, dissimilar or independent. Moran’s I is defined as follows [20]:

$$\text{I}=\frac{\text{n}\sum_{\text{i}=1}^{\text{n}}\sum_{\text{j}=1}^{\text{n}}{\text{w}}_{\text{ij}}({\text{x}}_{\text{i}}-\bar{\text{x}})}{{\text{s}}^{2}\sum_{\text{i}=1}^{\text{n}}\sum_{\text{j}=1}^{\text{n}}{\text{w}}_{\text{ij}}}$$ (5)

Where n is the total number of regions, xi denotes the observation value of the i-th region, and–x denotes the average value for all regions, and wij is the value of the spatial weight matrix defined above and is the sample variance. A spatial weight matrix for the 29 regions was first constructed, and then the Moran’s I was used to study the global spatiality. In this work, a 0–1 weighted distance matrix was used. The value is 1 if two regions are adjacent and 0 if they are not. Because Hainan is geographically an island, in terms of the neighboring relationship, Guangdong and Hainan were taken into consideration of each other’s neighboring regions in the process of constructing the spatial weight matrix.

### 4. Analysis for the spatial cluster pattern of health service development

Anselin (1995) proposed the local Moran’s I to test whether there is clustering of similar or dissimilar observation values in local regions. The local Moran’s I of region i is used to measure the relationship between region i and its neighbors, and defined as follows:

$${\text{I}}_{i}=\frac{\left(x_{i}-\bar{x}\right)}{S^{2}}\sum_{j\neq i}(x_{j}-\bar{x})$$ (6)

The local Moran’s I is used to present whether a specific region is correlated with its neighboring regions. The local Moran’s I also has a value between -1 and 1. When the local Moran’s I is greater than 0, it means that region i and its neighboring regions have similar characteristic properties, which indicates that they all have high level or low level development. On the contrary, when the local Moran’s I is less than 0, it means that region i and its neighboring regions have the opposite characteristic properties, which indicates that they are high-low cluster or low-high cluster. The spatial clusters of health service development in different regions were analyzed by calculating the local Moran’s I as defined above and drawing the local Moran’s I scatter plot. The scatter plot is used to show the correlation between the variables of a region and its neighboring regions by plotting the positions of different regions on the two-dimensional plane (Y, WY). The actual development level of health services is indicated by the spatial weight matrix and the spatial-lagged level of health service development.

## System construction and index selection

Measuring the effectiveness of health services in a region is also a part of the performance evaluation for the regional government, and this measurement can also help the government to better know the actual status of the current effectiveness of public health services in rural regions and improve its own service capability to ensure the well-being of local residents. So it is particularly important to measure the effectiveness of public health services. The scientific, comprehensive and reasonable efficiency of public health services can reflect the service effectiveness of a region. A scientific and objective evaluation system for the effectiveness of rural public health services should consist of a number of interrelated indexes that objectively reflect the dynamic rules of development of rural public health services. It is important to understand who is being evaluated, what is being evaluated, how it is being evaluated, and who is evaluating. On top of that, scientifically defining the range of evaluation indexes for the effectiveness of rural public health services is a prerequisite for scientifically evaluating health service. Evaluation content must take into account China’s current national and regional realities. The scope of the evaluation should not be too extensive, otherwise the content will be complicated, the workload will be heavy, the evaluation will be difficult to quantify, and the key elements will be difficult to identify. However, the scope of evaluation also should not be too small. An evaluation consisting only one or two contents regarding public health service is narrow and is difficult to make a comprehensive and scientific conclusion. Therefore, the indexes for evaluation should be in an appropriate range. The indexes should be quantifiable and with easily accessible data and meantime are selected from representative, referential and more stable parts of the public health services. This work investigated the situation of rural public health services from two aspects. On the basis of scientifically defining the relevant indexes and setting the score distribution, a relatively objective index reflecting the level of rural public health services in a region is constructed using input and output indexes.

Therefore, the measurement of the development of high-quality public medical and health services is a systematic project that needs to comprehensively consider the economy, society, ecological civilization, people’s livelihood and health. The development of high-quality public medical and health services is not only the pursuit of high-quality development process, but also the pursuit of high-quality development results. Grasp not only the current momentum of high-quality public medical and health services, but also grasp the prospects and potential of the overall and various parts of the service development. The performance of a local government in rural public health services is mainly shown in the input indexes such as the construction of infrastructures and clinics, the number of skilled health personnel and beds in rural health facilities per 1000 rural population. These indexes are directly related to the perception of people in the health services. Therefore, it is important to have indexes to show the capacity of public health services. In order to directly show the capacity of the government in public health services, the following output indexes are chosen for this work: 1) The hospital discharge rate, 2) The bed utilization ratio, 3) The average days in hospital, 4) Daily visits per doctor, 5) Daily inpatients per doctor. Therefore, this work constructed an evaluation index system for public health services in rural China with 10 input and output indexes, which are shown in Table 1.

**Table 1:** Evaluation index system for public health services in rural China.

Index classification	Index	Unit	Calculation
Input indexes	The number of clinics	piece	Number of village-level health institutions
	The number of skilled health personnel per 1000 rural population	person	Rural health technicians/population x1000
	The number of doctors (assistant) per 1000 rural population	person	(Number of licensed physicians + number of licensed assistant physicians)/population x100
	The number of registered nurses per 1000 rural population	person	Number of registered nurses/population x1000
	The number of beds in rural health facilities per 1000 rural population	piece	Number of beds/population in medical and health institutions x1000
Output indexes	The hospital discharge rate	%	Number of discharged/total number of admitted
	The bed utilization ratio	%	Actually occupied total bed days/actually opened total bed days x 100%
	The average days in hospital	Day/Person	Total number of bed days occupied by discharged persons/number of discharged persons
	Daily visits per doctor	frequency	Number of consultations/average number of doctors/251
	Daily inpatients per doctor	day	Actual number of bed days/average number of doctors/365

Based on the above basic assumptions about the inputs and outputs of rural public health services, a comprehensive service evaluation system for rural public health services is constructed. The comprehensive service performance of the government was evaluated with the local input and output indexes. In summary, this work follows the principle that the relevant indexes should be as quantifiable, comparable, more stable, more influential, and representative as possible in the selection of relevant indexes.

## Measurement of the level of rural public health services

### Data sources and processing

Based on the above evaluation index system, the relevant data of 29 regions (excluding Tibet, Hong Kong, Macao and Taiwan) of China from 2004 to 2018 were collected. Due to the lack of the data in rural regions of Beijing and Shanghai, Beijing and Shanghai were excluded. All data come from China Statistical Yearbook and China Health Statistics Yearbook.

## Results on the measurement of the level of rural public health services

Based on the China’s provincial panel data of 29 regions from 2004 to 2018, the DEA model was used to obtain the efficiency of rural public health services, and the details are shown in Tables 2 and 3.

**Table 2:** Efficiency of rural public health services from 2004 to 2010.

Region	2004	2005	2006	2007	2008	2009	2010	Average
Tianjin	1	1	1	1	1	1	1	1.000
Hebei	0.806	0.841	1	1	0.85	0.908	0.939	0.954
Shanxi	0.706	0.937	0.808	0.732	0.649	0.787	0.704	0.839
Inner Mongolia	0.842	0.717	0.728	0.735	0.7	0.734	0.831	0.764
Liaoning	0.702	0.809	0.687	0.686	0.705	0.711	0.772	0.818
Jilin	0.671	0.777	0.638	0.593	0.605	0.643	0.747	0.729
Heilongjiang	0.737	0.821	0.707	0.725	1	0.717	0.822	0.856
Jiangsu	1	1	1	1	1	1	1	0.929
Zhejiang	0.772	0.923	0.85	1	0.746	0.954	0.994	0.933
Anhui	1	1	1	0.992	0.999	1	0.986	0.998
Fujian	1	1	0.934	0.881	1	1	0.965	0.927
Jiangxi	0.959	0.903	0.92	0.882	0.914	0.905	0.954	0.962
Shandong	0.732	0.806	0.699	0.747	0.681	0.704	0.727	0.708
Henan	1	1	1	1	1	1	0.949	0.997
Hubei	1	0.881	1	1	0.9	1	0.957	0.939
Hunan	0.789	0.74	0.835	0.804	0.847	0.844	0.84	0.863
Guangdong	1	1	1	1	1	1	1	0.999
Guangxi	1	1	1	1	0.998	0.976	0.948	0.995
Hainan	1	1	1	1	1	0.962	1	0.997
Chongqing	1	1	1	1	1	1	1	1.000
Sichuan	0.89	0.876	0.945	0.826	0.815	0.847	0.841	0.892
Guizhou	1	1	1	1	1	1	1	0.997
Yunnan	0.989	1	1	0.905	0.928	0.994	1	0.988
Tibet	1	1	1	1	1	1	1	1.000
Shaanxi	0.673	0.75	0.75	0.844	0.74	0.932	0.821	0.891
Gansu	1	1	0.93	0.919	0.866	1	1	0.978
Qinghai	0.992	0.854	0.967	1	1	1	1	0.988
Ningxia	1	1	1	1	1	1	1	1.000
Xinjiang	1	1	1	1	1	0.836	1	0.984
Average	0.906	0.918	0.910	0.906	0.895	0.912	0.924	

**Table 3:** Efficiency of rural public health services from 2011 to 2018.

Region	2011	2012	2013	2014	2015	2016	2017	2018	Average
Tianjin	1	1	1	1	1	1	1	1	1.000
Hebei	0.998	1	1	1	1	1	1	0.961	0.954
Shanxi	0.789	0.844	0.82	0.877	0.937	1	1	1	0.839
Inner Mongolia	0.832	0.807	0.759	0.769	0.715	0.786	0.792	0.709	0.764
Liaoning	0.771	0.831	0.833	0.769	1	0.998	1	1	0.818
Jilin	0.711	0.705	0.673	0.861	0.748	0.909	0.883	0.768	0.729
Heilongjiang	0.886	0.888	0.867	0.934	0.924	0.922	0.95	0.939	0.856
Jiangsu	0.946	0.856	0.794	0.823	0.837	0.898	0.858	0.929	0.929
Zhejiang	1	1	0.94	0.964	0.997	1	0.929	0.92	0.933
Anhui	1	1	1	1	1	1	1	1	0.998
Fujian	0.888	0.922	0.886	0.872	0.876	0.909	0.886	0.881	0.927
Jiangxi	1	1	1	1	1	1	1	1	0.962
Shandong	0.623	0.61	0.632	0.635	0.72	0.739	0.768	0.791	0.708
Henan	1	1	1	1	1	1	1	1	0.997
Hubei	1	1	1	0.903	0.844	0.821	0.856	0.927	0.939
Hunan	0.903	0.906	0.928	0.948	0.91	0.892	0.908	0.857	0.863
Guangdong	1	1	1	1	1	1	1	0.979	0.999
Guangxi	1	1	1	1	1	1	1	1	0.995
Hainan	1	1	1	1	1	1	1	1	0.997
Chongqing	1	1	1	1	1	1	1	1	1.000
Sichuan	0.876	0.888	0.903	0.931	0.983	0.905	0.916	0.934	0.892
Guizhou	1	1	1	1	1	1	1	0.958	0.997
Yunnan	1	1	1	1	1	1	1	1	0.988
Tibet	1	1	1	1	1	1	1	1	1.000
Shaanxi	0.999	0.954	0.972	1	0.996	1	0.967	0.962	0.891
Gansu	1	1	1	1	1	1	0.993	0.966	0.978
Qinghai	1	1	1	1	1	1	1	1	0.988
Ningxia	1	1	1	1	1	1	1	1	1.000
Xinjiang	1	1	1	1	0.995	1	1	0.924	0.984
Average	0.939	0.938	0.931	0.941	0.948	0.958	0.955	0.945	

The efficiency value calculated by the model is within the interval [0, 1], the closer it gets to 1, the higher the efficiency. If it equals to 1, then the DEA is efficient. The above tables demonstrate that the great majority of regions in China show a low efficiency in the rural public health services from 2004 to 2018. In addition, the data reveal that only Tianjin, Chongqing, Tibet and Ningxia show an average efficiency value equal to 1, reflecting the efficient rural public health services, which accounts for 13% of the total. In other words, the service efficiency values of all the other regions are less than 1. The average efficiency value of rural public health services on our collected data is 0.928. 18 regions exceed the average value, which accounts for 62% of the total regions, reflecting the weak efficiency in the rural public health services. The remaining 11 regions show inefficiency and a low level in the rural public health services. The above result indicates a huge disparity in the service efficiency between regions. China’s rural public health services still have great potential for improvement. In terms of orientation, the average efficiency values are 0.918, 0.898, 0.956 in the eastern, central and western regions, respectively. The service efficiency value of the western region is significantly higher than that of the eastern and central regions and the national average, showing the spatial characteristics that the service efficiency is high in the eastern and western regions but low in the central region. This result is attributed to the fact that the eastern coastal regions have more complete health infrastructures and better qualified human resources, and the western regions have been receiving attention in recent years and continued to vigorously develop its economy and infrastructures to improve its service capacity. Therefore both the eastern and western regions show a higher efficiency value in the health service. In terms of regions, the regions that have a service efficiency value higher than the national average are in a proportion of 62%, indicating that the efficiency of health services has been further improved in most regions. The four regions with the highest average efficiency are Tianjin, Chongqing, Tibet, and Ningxia in order, and three of which are in the western regions. The five regions with the lowest average efficiency are Shandong, Jilin, Inner Mongolia, Liaoning, and Shanxi in order, and most of them are in the central region.

### Temporal distribution of rural public health service efficiency

**Fig 1:**
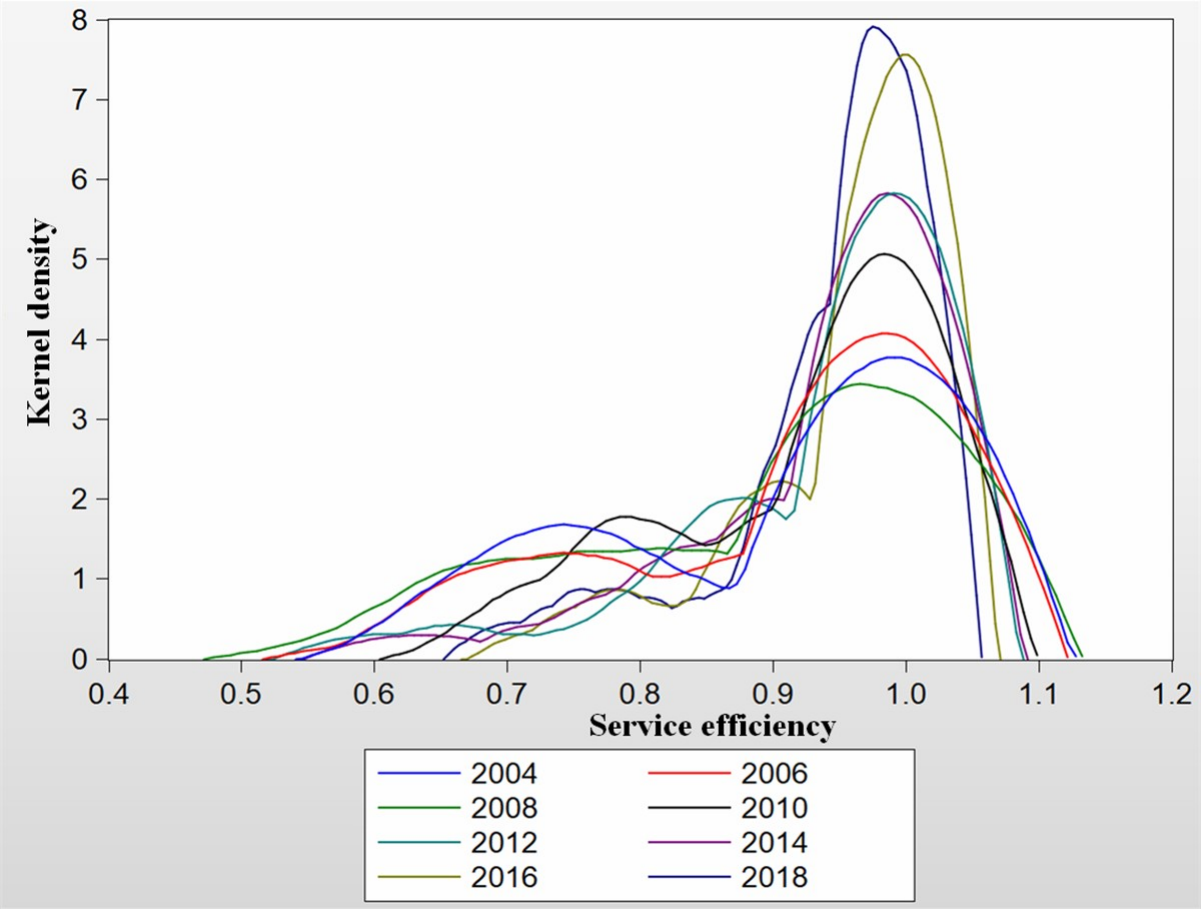
Kernel density distribution for China’s rural public health services efficiency from 2004 to 2018.

In this work, the kernel density estimation method was used to study the peak and distribution changes of China’s rural public health services efficiency and to analyze its dynamic evolution characteristics. Eviews8 software was applied to the kernel estimation of China’s rural public health services efficiency, and a two dimensional graph of the kernel density was obtained and shown in Fig 1. The typical years 2004, 2006, 2008, 2010, 2012, 2014, 2016 and 2018 were selected to draw the kernel density curves. The service efficiency is based on the results obtained from DEA method, and the Kernel density which is in essence probability density mainly helps to compare and reference. It is worth noticing that the Kernel density and service efficiency, which are vertical axis and horizontal axis labels respectively, have no units owing to nondimensionalization. Comparing the curves of different years, the temporal dynamic evolution characteristics of China’s rural public health services efficiency can be obtained.

In the curves, the change of the central position of the density function in the 8 years is not obvious. With an increase of the year, there is a tendency to gradually shift the kernel density distribution curve to the right. After 2010, the extent of the shift is slightly increased, indicating that the health service efficiency has a greater increase after 2010. In short, China’s regional health services have been slowly improving. From the view of the shapes of the curves, the 4 years on the left side of Fig 1 have smaller changes, but the years on the right side have the opposite trend. In 2004, 2006, 2008, and 2012, there are great differences in the curves of these 4 years, indicating a large gap in the development between regions. The shapes of the whole distribution curves show a steep first and then gentle trend, and also reveal a low concentration. From the view of the density value, the height of the main peak rises first and then declines, and the width of the main peak gradually becomes wider, which indicates that the polarization trend of the efficiency of the rural health services in China has been gradually weakening. During the observation period, the main peak value of the kernel density curve increases significantly, but the small peak value tends to decrease. This result means that the gap between the regions with low service efficiency in China is gradually increasing, but the gap between the regions with high services efficiency is decreasing. The interregional uncoordinated development is obvious. From 2004 to 2018, the center of the kernel density function did not change significantly. The highest peak value decreases year by year, and these peaks are wide. This result indicates that the distribution of the efficiency of health services is scattered. The wave of the kernel density curve shifts to the right, the vertical height of the peak decreases, and the horizontal width increases, which indicates that the index is increasing and the regional disparity becomes greater. This result means that from the view of the number of peaks, the kernel density distribution of China’s service efficiency has always shown the characteristics of a “scattered double peaks” model. The double peaks which were shown in all the curves indicate an uncoordinated development in China’s ecological efficiency. Furthermore, the polarization of the interregional coordination index has always existed.

### Spatial correlation analysis on rural public health services

Stata14.0 was used to calculate the Moran’s I for the health service development in various regions of China from 2004 to 2018, as shown in Table 4. Clearly, the P values of Moran’s statistic for the health service development from 2004 to 2018 are all less than 0.1, indicating that the development of China’s rural public health services has a significant spatial correlation in the entire regions. In terms of geographical location, there is a strong spatial dependency effect on the development of health services in different regions. The development of health services of a region is not only related to its own development, and is also positively affected by the neighboring regions, which is attributed to the mutual resource flows between regions, indicating the spatial spillover effect between regions in the public health services.

**Table 4:** Global Moran’s I.

Year	I	P
2004	0.1253	0.0923
2006	0.1861	0.0393
2008	0.1687	0.052
2010	0.1415	0.066
2012	0.1289	0.073
2014	0.1247	0.084
2016	0.1275	0.079
2018	0.1281	0.0832

Moran’s scatter plot has four quadrants, where the first and third quadrants refer to positive spatial correlation, and the second and fourth quadrants refer to negative spatial correlation. The specific meaning of each quadrant is as follows: 1) The first quadrant refers to that the regions and their neighboring regions have a high development level in health services, which indicates that the regions with well-developed public health services are adjacent to the regions with well-developed public health services, showing a high-high cluster. This quadrant is denoted as H-H.

**Fig 2:**
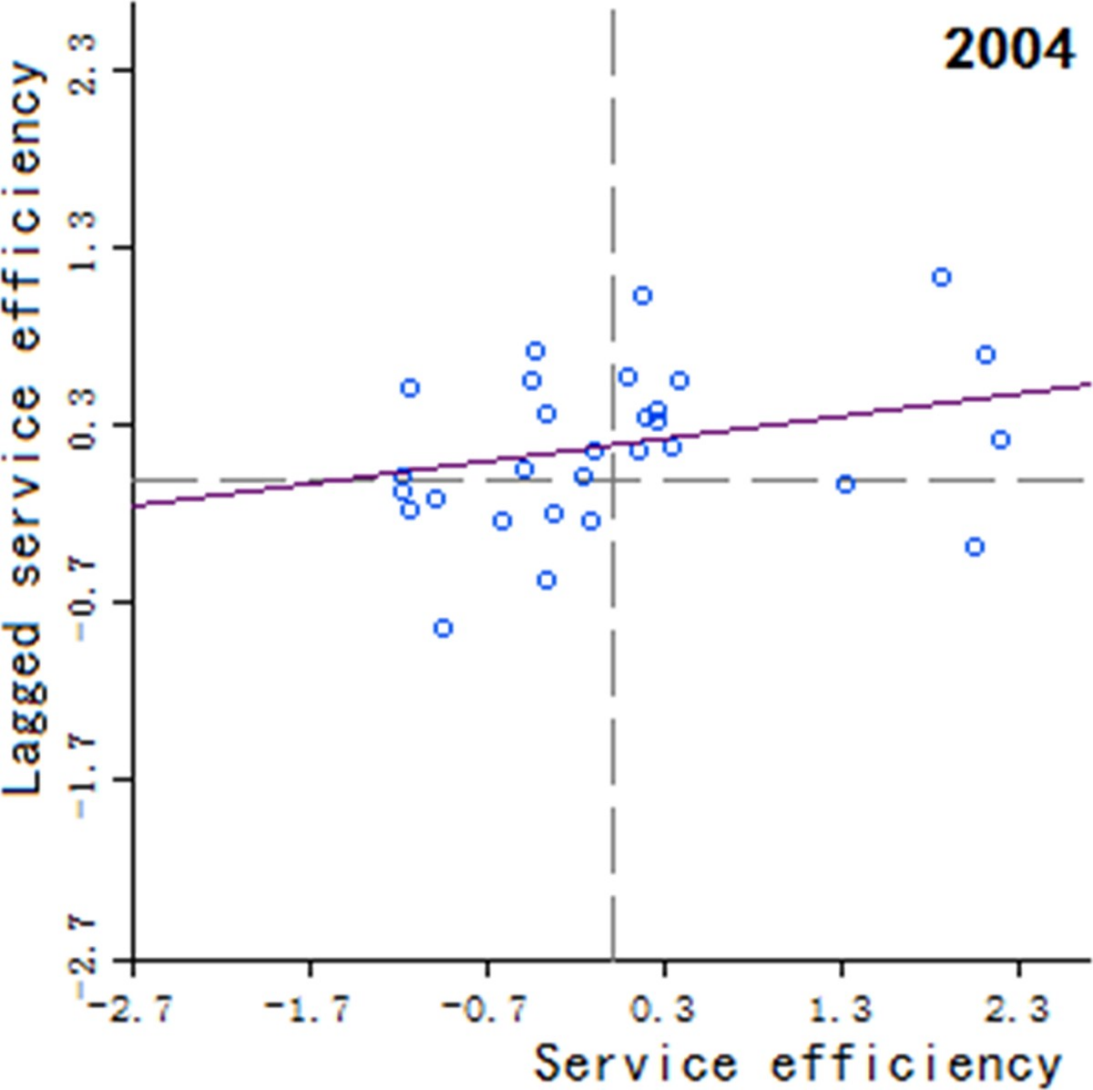
Scatter plot of China’s regional medical and health service level distribution from 2004 to 2018.

**Fig 3:**
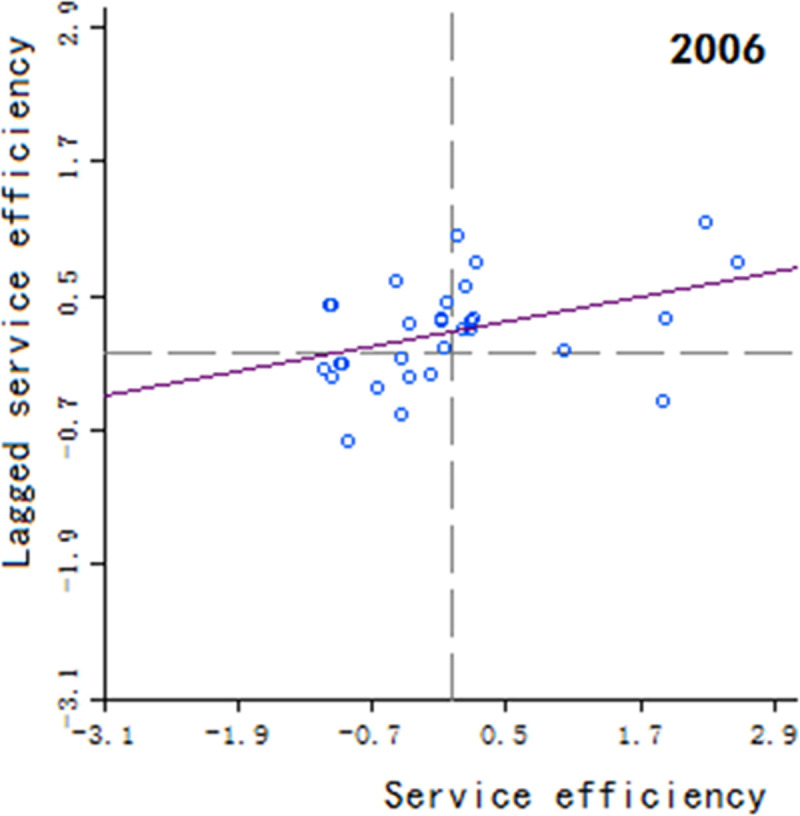
Scatter plot of China’s regional medical and health service level distribution from 2004 to 2018.

**Fig 4:**
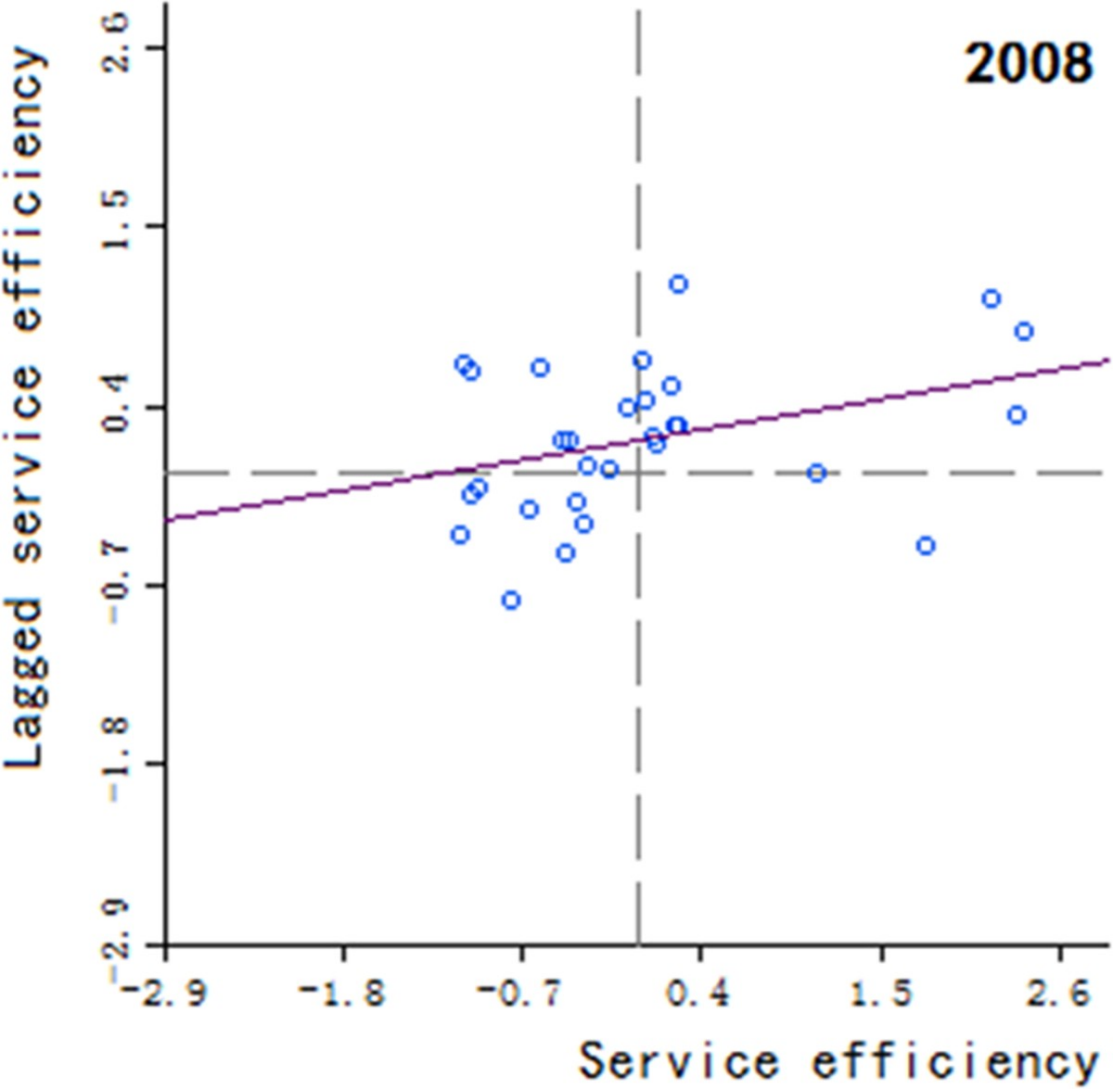
Scatter plot of China’s regional medical and health service level distribution from 2004 to 2018.

**Fig 5:**
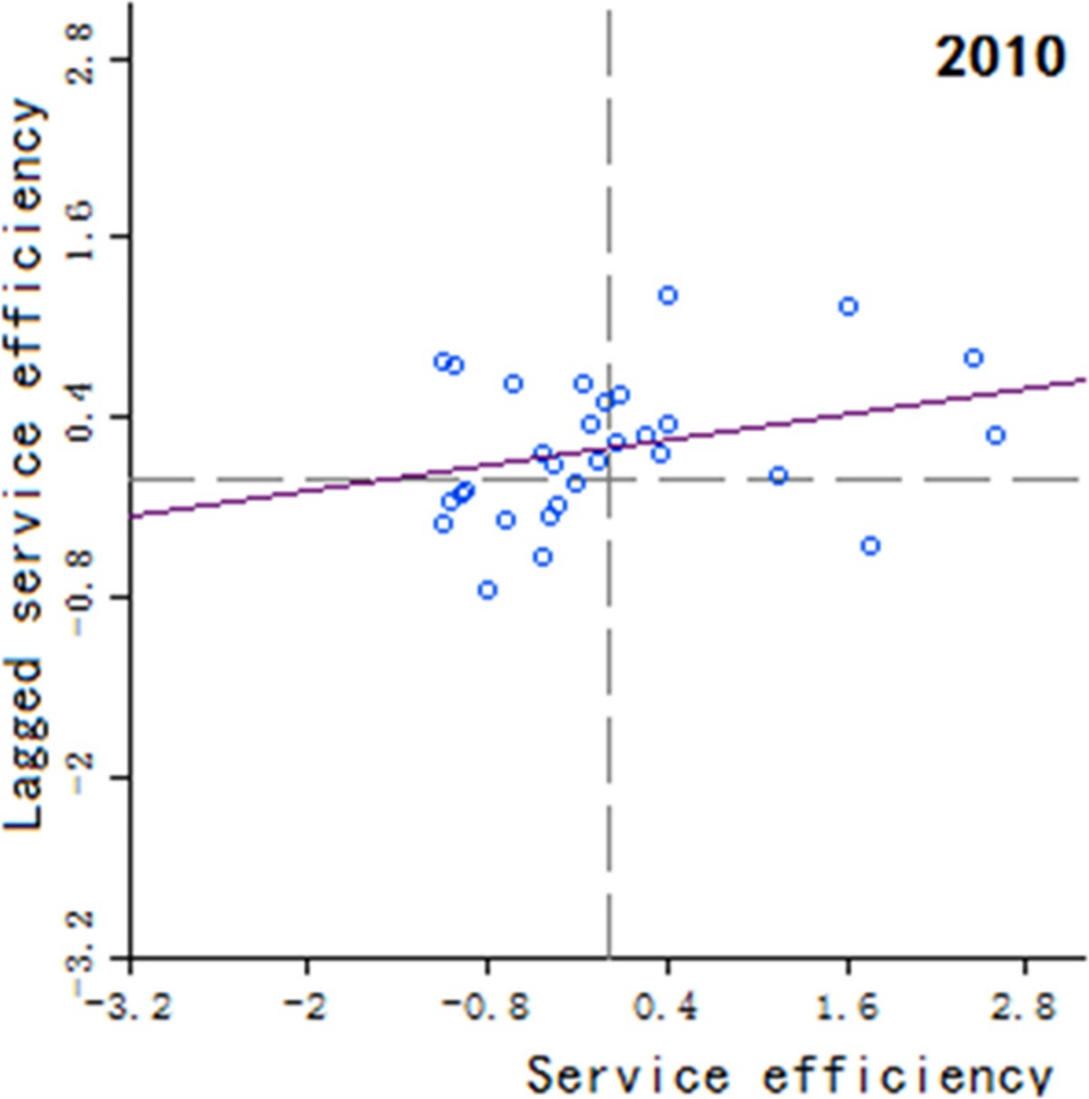
Scatter plot of China’s regional medical and health service level distribution from 2004 to 2018.

**Fig 6:**
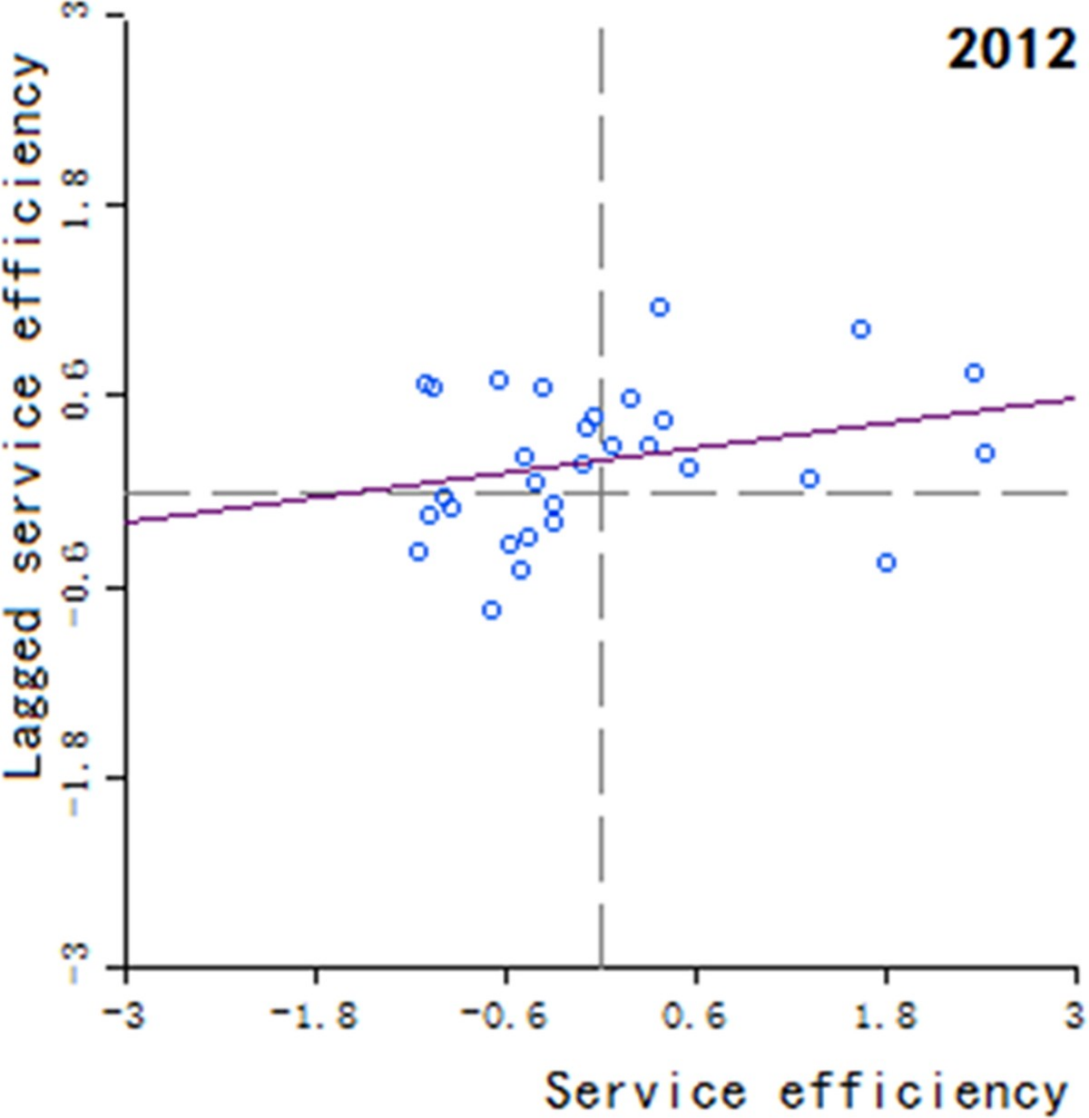
Scatter plot of China’s regional medical and health service level distribution from 2004 to 2018.

**Fig 7:**
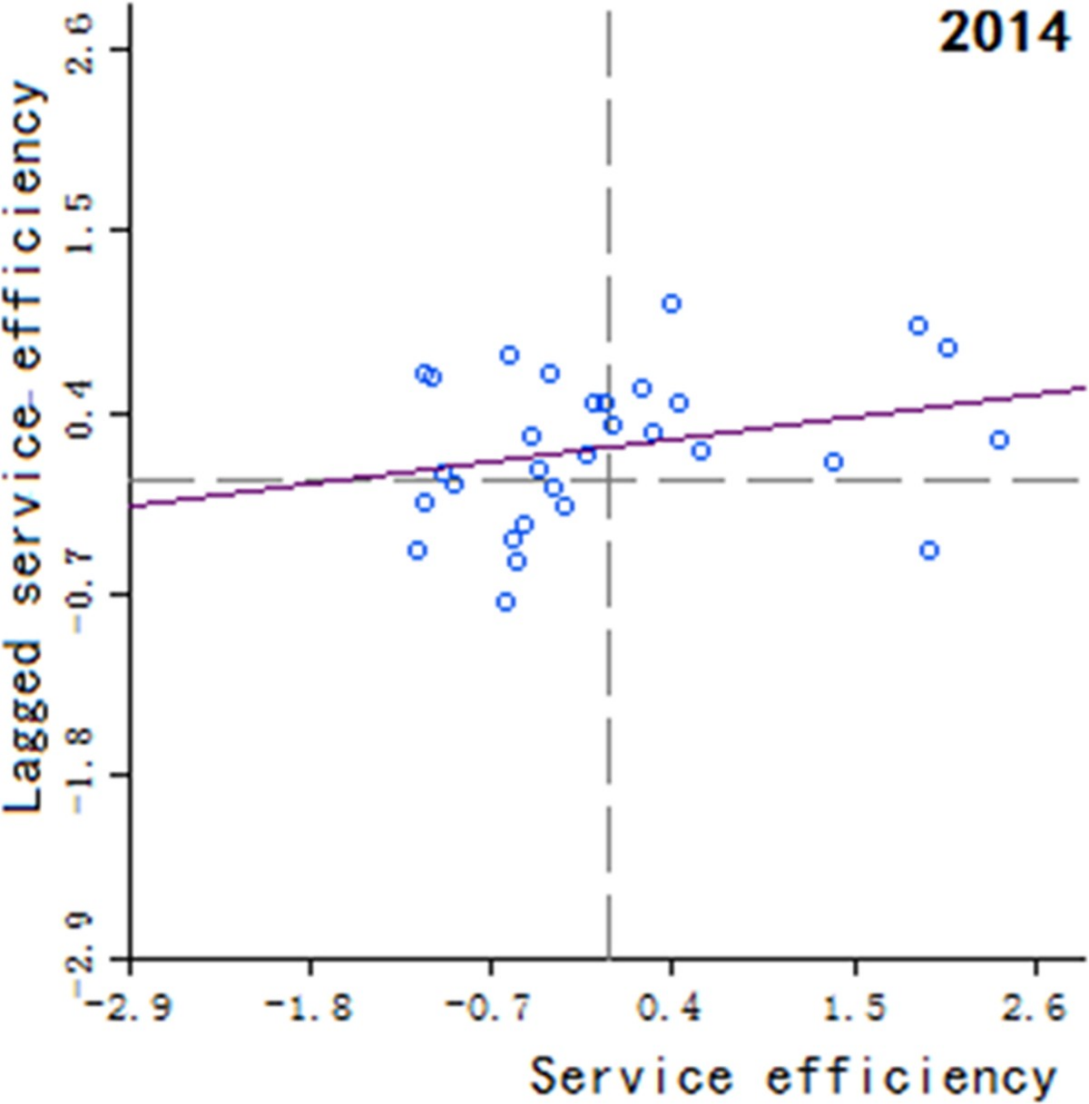
Scatter plot of China’s regional medical and health service level distribution from 2004 to 2018.

**Fig 8:**
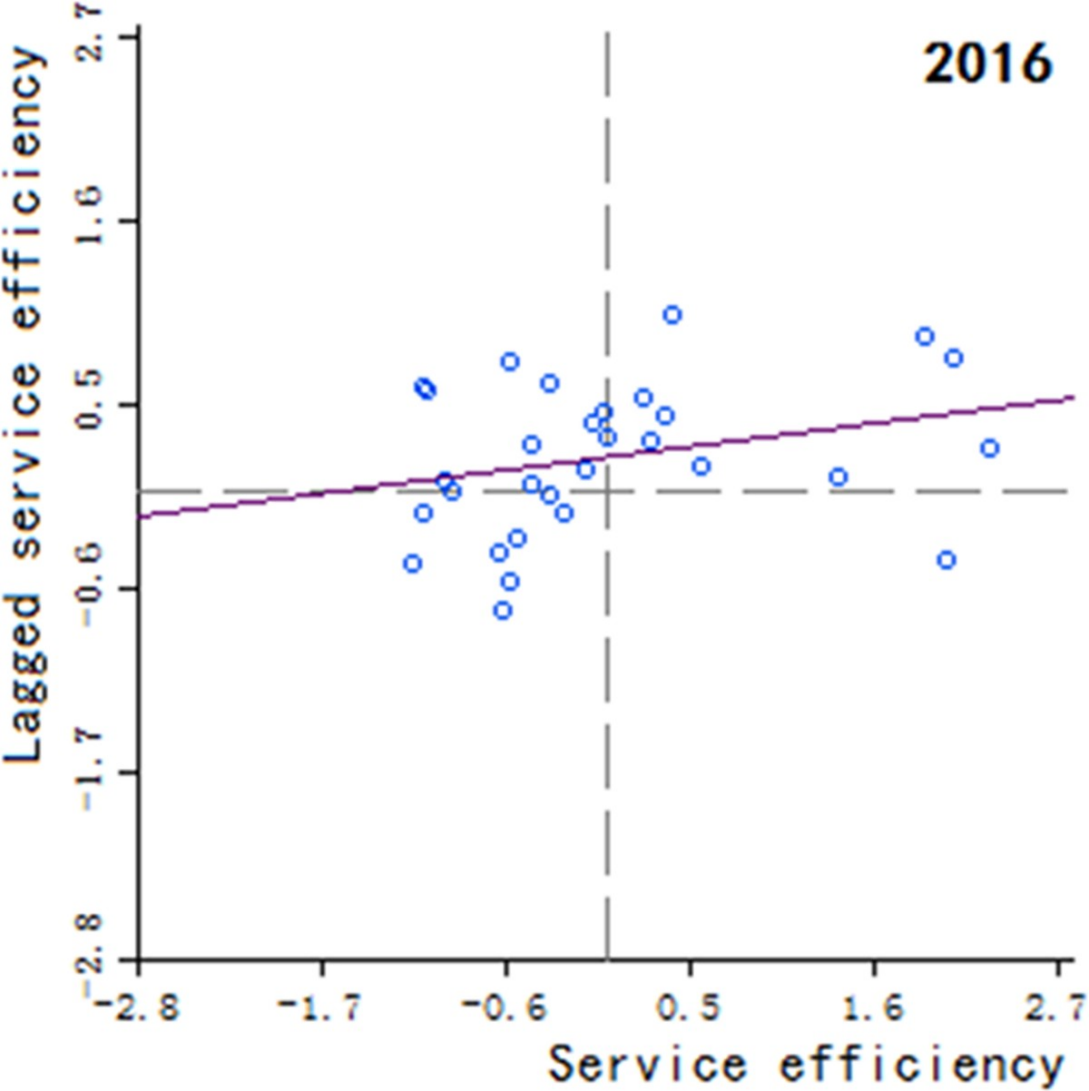
Scatter plot of China’s regional medical and health service level distribution from 2004 to 2018.

**Fig 9:**
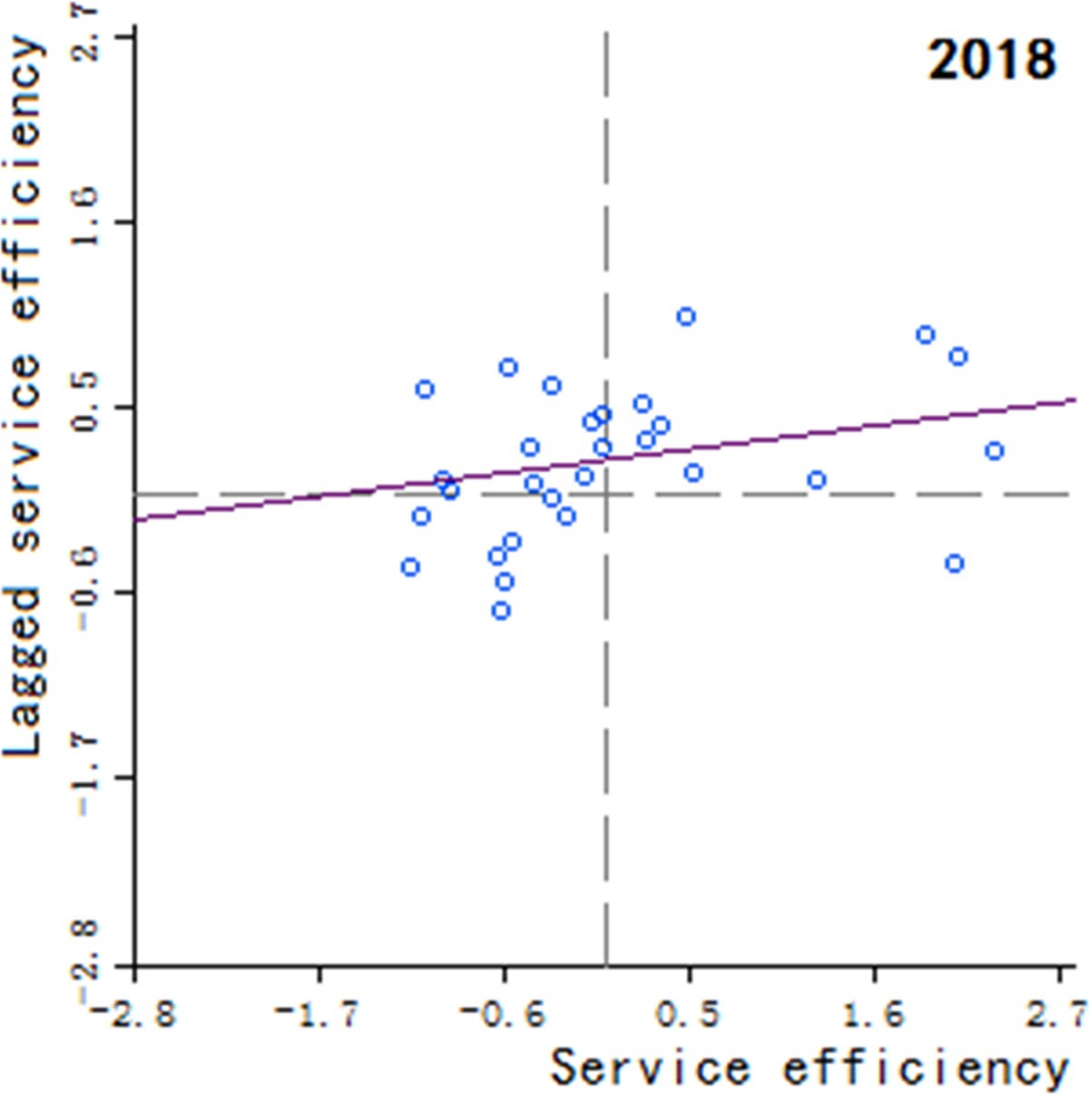
Scatter plot of China’s regional medical and health service level distribution from 2004 to 2018.

2) The second quadrant refers to that the regions have a low development level in health services, but their neighboring regions have a high development level in health services, showing a low-high cluster. This quadrant is denoted as L-H. 3) The third quadrant refers to that the regions and their neighboring regions have a low development level in health services, showing a low-low cluster. This quadrant is denoted as L-L. 4) The fourth quadrant refers to that the regions have a high development level in health services, but their neighboring regions have a low development level in health services, showing a high-low cluster. This quadrant is denoted as H-L, shown in Figs 2–9.

Based on the above description, the corresponding results for the Moran’s scatter plot are listed in detail in Table 5. The distributions of the regions for the development of health services for nearly 15 years in the four quadrants are almost the same. Specifically, the regions in H-H are mainly Hebei, Shanxi, Jiangxi, Shandong, Henan, Hubei, Hunan, Guangdong and Shaanxi. These regions generally have a high development in public health services and are mostly from the regions with relatively developed economic in the center of China. Therefore, they show a high-high cluster. The regions in L-H are mainly Inner Mongolia, Liaoning, Anhui, Fujian, Chongqing, Guizhou, Yunnan, Qinghai. These regions have a low development in health services, but their neighboring regions have a high development in health services. The region in H-L is almost only Sichuan, which indicates that Sichuan itself has a high development in public health services, but the neighboring regions such as Tibet, Xinjiang, Qinghai and Gansu have a lower development, resulting in a high-low cluster. The rest of the regions are in the third quadrant. Most of them are from western, central and northeastern regions. They have a low development in health services, and at the same time the neighboring regions also have a low development in health services, showing a low-low cluster.

**Table 5:** Cluster of the development of public health services.

Quadrants	2018	2016	2014	2012	2010	2008	2006	2004
H-H	Hebei	Hebei	Hebei	Hebei	Hebei	Hebei	Hebei	Hebei
	Shanxi	Shanxi	Shanxi	Shanxi	Shanxi	Shanxi	Shanxi	Shanxi
	Jiangxi	Jiangxi	Jiangxi	Jiangxi	Jiangxi	Liaoning	Anhui	Liaoning
	Shandong	Shandong	Shandong	Shandong	Shandong	Anhui	Jiangxi	Jiangsu
	Henan	Henan	Henan	Henan	Henan	Jiangxi	Shandong	Anhui
	Hubei	Hubei	Hubei	Hubei	Hubei	Shandong	Henan	Shandong
	Hunan	Hunan	Hunan	Hunan	Hunan	Henan	Hubei	Henan
	Guangdong	Guangdong	Guangdong	Guangdong	Guangdong	Hubei	Hunan	Guangdong
	Shaanxi	Guangxi	Guangxi	Guangxi	Guangxi	Guangdong	Guangdong	Guangxi
		Shaanxi	Shaanxi	Shaanxi	Shaanxi	Guangxi	Guangxi	Guizhou
						Shaanxi	Shaanxi	Shaanxi
L-H	Tianjin		Tianjin	Tianjin	Tianjin	Tianjin	Tianjin	Tianjin
	Inner MongoliaLiaoning	Tianjin	Inner MongoliaLiaoning	Inner MongoliaLiaoning	Inner MongoliaLiaoning	Inner Mongolia	Liaoning	Inner Mongolia
	Guangxi	Inner MongoliaLiaoning	Anhui	Anhui	Anhui	Jiangsu	Jiangsu	Fujian
	Anhui	Anhui	Fujian	Fujian	Fujian	Fujian	Fujian	Jiangxi
	Fujian	Fujian	Chongqing	Chongqing	Chongqing	Chongqing	Chongqing	Hubei
	Chongqing	Chongqing	Guizhou	Guizhou	Guizhou	Guizhou	Guizhou	Chongqing
	Guizhou	Guizhou	Yunnan	Yunnan	Yunnan	Yunnan	Yunnan	Yunnan
	Yunnan	Yunnan	Qinghai					
	Tibet	Qinghai						
	Qinghai							
L-L	Jilin	Jilin	Jilin	Jilin	Jilin	Jilin	Inner Mongolia	Jilin
	Heilongjiang	Heilongjiang	Heilongjiang	Heilongjiang	Heilongjiang	Heilongjiang	Heilongjiang	Heilongjiang
	Jiangsu	Jiangsu	Jiangsu	Jiangsu	Jiangsu	Zhejiang	Zhejiang	Zhejiang
	ZhejiangGansu	ZhejiangTibet	Zhejiang	Zhejiang	Zhejiang	Tibet	Tibet	Tibet
	Ningxia	Gansu	Tibet	Tibet	Tibet	Gansu	Gansu	Gansu
	Xinjiang	Ningxia	Gansu	Gansu	Gansu	Qinghai	Qinghai	Qinghai
		Xinjiang	Ningxia	Qinghai	Qinghai	Ningxia	Ningxia	Ningxia
			Xinjiang	Ningxia	Ningxia	Xinjiang	Xinjiang	Xinjiang
				Xinjiang	Xinjiang			
H-L	Sichuan	Sichuan	Sichuan	Sichuan	Sichuan	Sichuan	Sichuan	Sichuan
						Hunan		

## Conclusion and discussion

In this work, the development of China’s rural public health services was studied. The evaluation index system for China’s rural public health services was constructed. The DEA method was used to measure the level of rural public health services in 29 regions of China from 2004 to 2018. And then the efficiency of China’s public health services was analyzed from the perspectives of spatiotemporal dynamic distribution and evolutionary trend. The **conclusions** are summarized as follows:

1. The health services in the areas with low supply levels have a relatively faster development. This is mainly due to the fact that the policies and measures adopted by the government to promote the equalization of basic public health services, such as strengthening the construction of public health institutions and guaranteeing the funding of public health services, can be a useful supplement to the areas with relatively poor health resources, while the effect is not obvious for the areas with relatively rich health resources. As a result, the efficiency of rural health services in China is growing slowly. There is a large and increasing gap between regions in the efficiency, which indicates an uncoordinated development between regions. And the trend towards multi-polarity has long existed.
2. The service efficiency shows the spatial distribution characteristics of “high in the center and low in the eastern and western regions”. The proportion of the regions with high service efficiency shows a downward trend. The number of the regions with high and middle service efficiency shows a fluctuating upward trend, most of them are in the southeast of China. The number of the regions with middle service efficiency has increased significantly, and they are distributed in the northeastern and central regions of China. The number of the regions with middle and low service efficiency is always the greatest, and most of them are in the center of China. The number of the regions with low service efficiency is more stable, and most of them are in the northwest of China.
3. There are some characteristics in the evolution of service efficiency. Level transitions are mostly occurred at adjacent levels. The service efficiency level generally shifts to the next lower level. The probability for cross-level transition is extremely low. In addition, the probability of upward transition is generally lower than the probability of downward transition. The level transition at higher levels is more fluctuated, while the level transition at lower levels is smoother and more persistent.
4. There is a large gap in the level of health expenditure between regions, and the health resources cannot cross the gap of social welfare, and the mobility of resource elements is poor. Therefore, this paper suggests that the government should change its functions, break the administrative system barriers, and create conditions for the free flow of health resources to realize the coordinated development of health services between regions and between urban and rural areas.

Based on the findings of the study, the following discussions are proposed:

1. Although the level of public health services in China is not uniform, after years of development, the difference in the level of public health services in the three regions has reduced, especially in the central and western regions. This result indicates that the strategy of China which aims to achieve the equalization of the level of public health services in various regions has achieved initial success.
2. To achieve the goal of narrowing the regional disparities in public health expenditure, fiscal policy focuses on two aspects: On the one hand, China should further accelerate the reform of the fiscal system, promote economic development in backward areas through incentive fiscal policies, and expand the scale of regional economies, which enhances the hard power in providing public health services. On the other hand, the central government’s investment in public health care needs to further support the underdeveloped regions. The public health investment in the central and western regions would continue to increase through transfer payments. To a certain extent, it helps to overcome the regional differences in the level of public health services caused by economic differences between regions.
3. In addition to what has been studied in this paper, there are actually further directions worthy of research. This paper focuses on the research of efficiency measurement and spatiotemporal disparity of rural public health services in China. Beyond that, future research can start from different aspects, such as the differences of public health services in China and abroad and the reasons for the differences, which helps to find the way to promote public health services in China and abroad. Besides, this paper focuses on quantitative research in terms of research methods. In the future, more complex simulation analysis can be used to make more comprehensive and in-depth research on how multiple subjects interact with each other in public health services.
